# Red Tide Detection Method Based on a Time Series Fusion Network Model: A Case Study of GOCI Data in the East China Sea

**DOI:** 10.3390/s25113455

**Published:** 2025-05-30

**Authors:** Tianhong Ding, Zhiqiang Xu, Yunjie Wang, Qinglian Hou, Xiangyong Liu, Fengshuang Ma

**Affiliations:** 1 The Fishery Machinery and Instrument Research Institute, Chinese Academy of Fishery Science, Shanghai 200092, China; 2 East China Sea Fisheries Research Institute, Chinese Academy of Fishery Sciences, Shanghai 200090, China

**Keywords:** red tide, NDVI, time-series samples, ASPC-DSC, CSF-RTDNet, ConvLSTM

## Abstract

In China’s coastal regions, severe seawater eutrophication has led to frequent occurrences of red tides, causing significant damage to marine fisheries and aquatic resources. Therefore, red tide detection and prediction are of great research importance. Although current deep learning-based red tide detection methods perform well in detecting single-day red tides, they struggle with continuous multi-day detection due to insufficient mining of temporal features and difficulties in accurately capturing dynamic variations, limiting further improvements in detection accuracy. To address these issues, this study proposes a time-series fusion network model (CSF-RTDNet) for red tide detection using time-continuous GOCI data from the East China Sea. By integrating multi-temporal GOCI data, the model comprehensively captures spatiotemporal characteristics of red tides, enhancing dynamic process modeling. The CSF-RTDNet method improves feature discrimination by introducing NDVI to enhance red tide characteristics and increase separability between red tides and seawater. Additionally, an ECA channel attention mechanism is employed to fully exploit spectral features across different bands for deeper feature extraction. A novel feature extraction module, ASPC-DSC, combines atrous spatial pyramid convolution with depthwise separable convolution to effectively fuse multi-scale contextual features while improving computational efficiency. Furthermore, ConvLSTM is introduced to integrate temporal and spatial features, effectively addressing the insufficient mining of sequential characteristics in multi-day red tide detection. Experimental results demonstrate that CSF-RTDNet achieves robust detection of red tides with complex boundaries and continuous temporal patterns, attaining an accuracy of 95.89%, precision of 93.03%, recall of 96.34%, and a Kappa coefficient of 0.95. This method significantly enhances red tide detection accuracy and provides valuable technical support for marine environmental monitoring.

## 1. Introduction

Red tide is a marine ecological phenomenon characterized by the excessive proliferation of phytoplankton, such as algae, in seawater, leading to the water turning red or other colors [1]. With the changes in the marine environment along China’s coast, the frequency of red tides has been increasing. Between 2011 and 2017, a total of 212 red tide events occurred in the East China Sea, with 6, 56, and 143 events in the waters off Shanghai, Fujian, and Zhejiang, respectively. Except for two large-scale red tides in Shanghai in 2016, Zhejiang experienced the highest frequency of red tides and the most extensive affected areas. The scale of red tides and the resulting economic losses have been growing, leading to increased negative impacts on marine ecosystems, coastal aquaculture, and the ecological environment. The toxins produced by red tides also pose a threat to human health [2]. In recent years, with the acceleration of industrialization and urbanization in China, a large amount of pollutants have been discharged into the ocean, exacerbating the deterioration of the marine environment and further contributing to the occurrence of red tides. In recent years, red tide disasters have attracted increasing attention, making red tide detection an important area of research.

In recent years, China has made significant progress in red tide detection. Traditional monitoring technologies utilizing platforms such as ships and satellites, combined with ground observations and laboratory analyses, have been employed to monitor and study the occurrence, development, and dissipation of red tides. Currently, there are three main traditional methods for detecting red tides: “visual observation”, “instrumental measurement”, and “remote sensing”. The first two methods require substantial human and material resources for red tide detection, and the harsh field conditions increase the difficulty of detection. Remote sensing, which uses satellites to detect red tides, is an advanced method established with modern scientific technology. This method relies entirely on instruments for observation and can provide timely, synchronous, and large-scale monitoring of red tides. Remote sensing methods for red tide detection are primarily divided into three categories. The first category involves using parameters such as chlorophyll content to identify red tides [3,4]. In 2018, Jiang Dejuan and colleagues used chlorophyll concentration and other parameters to visually identify red tides in the Bohai Sea [5]. The second category detects red tides by observing significant spectral differences in water bodies during red tide events. The explosive proliferation or aggregation of protozoa or bacteria during red tides causes changes in water color and spectral characteristics, particularly noticeable fluctuations around the green light band (550 nm). Remote sensing detects these spectral changes to identify red tides [6,7]. In 2017, Jiang Binbin and colleagues used the normalized water-leaving radiance at 550 nm as a characteristic band for red tide detection. The third category involves detecting red tides through the fluorescence of algae. For example, some algae exhibit fluorescence in the red light band [8]. In 2018, Zhang Feng and colleagues used the fluorescence line height of different water bodies to extract red tide indices, thereby determining the state of the water for red tide detection [9]. In 2021, Wang Siyuan and colleagues selected band information at 460, 530, 650, and 750 nm for red tide detection. Traditional red tide detection methods, based on expert experience, prior knowledge, and qualitative analysis of extensive reports, often have inaccurate and less applicable thresholds, limiting the improvement of red tide detection accuracy.

Deep learning has been widely applied in remote sensing applications such as image classification, target recognition, and image fusion. Due to its powerful capabilities in big data mining and feature extraction, deep learning has also been employed in red tide detection, achieving promising results [10,11]. This paper aims to detect red tides using satellite remote sensing image data, with image classification as the primary task. In China, with the continuous development of deep learning technology, an increasing number of scholars have begun to apply it to red tide detection. For instance, deep learning models like convolutional neural networks (CNN) have been used to identify red tides in satellite remote sensing images. By training models to extract features from red tide images, automatic identification and monitoring of red tides can be achieved. In 2018, Hu et al. proposed a CNN network composed of eight fully connected layers, demonstrating higher accuracy in red tide detection compared to traditional machine learning methods [12]. In 2019, Kim et al. utilized GOCI data and the U-Net network to study red tide detection in waters around the Korean Peninsula [13]. U-Net, widely used for pixel classification or target segmentation, has shown good performance in red tide detection. In 2022, Zhao et al. proposed the RDU-Net network based on the HY-1D coastal imager, improving the detection accuracy of red tide edges and dispersed distribution areas using deep learning. These studies validate the feasibility of using deep learning for remote sensing-based red tide detection [14]. In 2023, Ding et al. employed GOCI data and an improved U-Net network to study red tide detection in waters around the East China Sea, achieving effective detection of red tides on the day of occurrence and obtaining good detection results [15]. Overall, deep learning-based red tide detection and prediction technologies are still in a phase of continuous development and exploration both domestically and internationally. Although some progress has been made, there are still challenges and issues to be addressed. For example, deep learning models based on remote sensing information, such as CNN, have shown high detection accuracy in concentrated red tide areas but perform less ideally in detecting dispersed red tides. On the other hand, U-Net-based methods have demonstrated good performance in detecting large-scale concentrated and strip-distributed red tides and can effectively detect red tides on the day of occurrence. However, the detection accuracy for time-series red tide samples still needs improvement. Based on the above research, this paper proposes a red tide detection method based on a time series fusion network model.

## 2. Data and Methodology

### 2.1. Satellite Data

**Figure 1:**
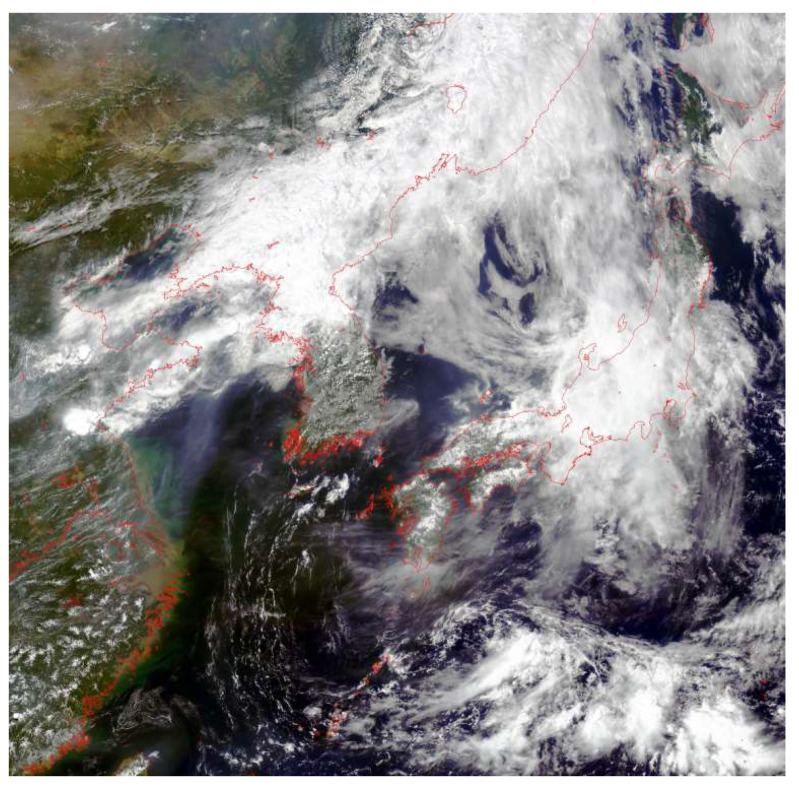
True color map of GOCI satellite coverage range.

The experimental data in this study were obtained by downloading corresponding data from the GOCI (Geostationary Ocean Color Imager) official website (Korea Ocean Satellite Center, Ansan, South Korea) based on red tide disaster information published in the China Marine Bulletin (Ministry of Natural Resources, Beijing, China). Currently, the main satellite sensors used include MODIS and GOCI. The GOCI satellite sensor can capture 8 images per day at 1-h intervals, unlike traditional polar-orbiting satellites that can only pass over a scene once per day. This makes it possible to monitor marine environments and marine disasters hourly through remote sensing. GOCI (Geostationary Ocean Color Imager) is a next-generation ocean color imager from South Korea, mounted on the COMS (Communication, Ocean, Meteorological Satellite), the country’s first geostationary meteorological satellite launched in 2010. It covers an area of 2500 km × 2500 km with a spatial resolution of 500 m and a very high temporal resolution, providing observational data from 8:30 to 15:30 over an eight-hour period at 1-h intervals. Figure 1 shows a true-color image of the coverage area of the GOCI satellite. The GOCI remote sensing images contain 8 bands: 412, 443, 490, 555, 660, 680, 745, and 865 nm, as detailed in Table 1. GOCI data are categorized into L1B, L2, and L3 levels based on processing stages.

### 2.2. Data Set Construction

The experimental data in this study were obtained by downloading corresponding data from the GOCI official website based on red tide disaster information published in the China Marine Bulletin. According to the red tide disaster information released in the China Marine Bulletin, a single large-scale red tide caused by Rhizosolenia setigera occurred in the waters east of Zhujiajian Island, Zhoushan, from August 14 to August 18, 2020. By screening true-color images with minimal cloud cover, GOCI L1B level data from August 14 to August 18, during the red tide outbreak in the East China Sea, was selected. The atmospheric correction of GOCI reflectance data was performed using the GOCI data atmospheric correction method provided by NASA OBPG’s official SeaDAS software. Subsequently, chlorophyll products were visualized to determine the approximate range of the red tide. A region with complete data was then extracted, and the red tide index for each pixel was calculated based on the red tide index formula. The red tide affected areas were delineated by combining chlorophyll concentration, the red tide index, and the red tide disaster information published in the China Marine Bulletin. Labels were created for both normal water bodies and red tide disaster areas [16].

The Red Tide Index (RI) was proposed by Ahn and Shanmugam in 2006 as an evaluation metric to identify potential algal blooms in the optically complex coastal waters of Northeast Asia. In 2014, Lou et al. revised the original RI by using remote sensing reflectance (Rrs) values at 443, 490, and 555 nm bands. The revised RI operates on the same principle as calculating chlorophyll-α concentration using the ratio of blue-green spectral bands. Formula (1) is as follows:

(1) $$RI=\frac{R_{rs}\left(555\right)-R_{rs}\left(443\right)}{R_{rs}\left(490\right)-R_{rs}\left(443\right)}$$

**Figure 2:**
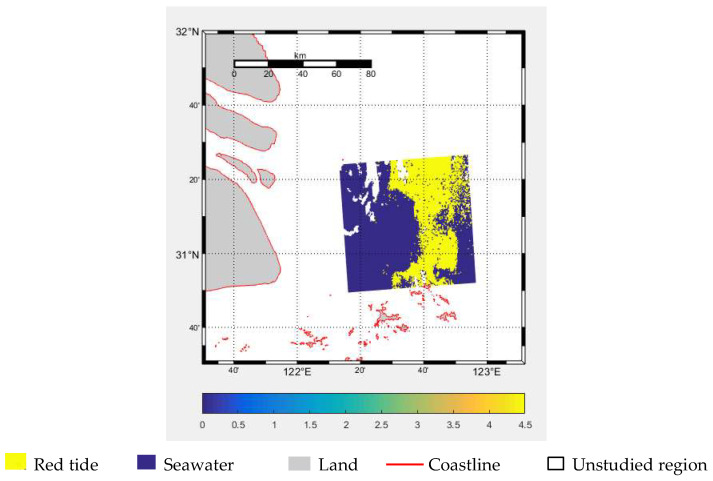
Red tide index visualization results.

The study area is primarily used for the design and validation of the model algorithm. Due to extensive cloud cover during certain periods and to ensure sample continuity and balance, remote sensing data from 13:30 on the 14th to the 17th were selected. Additionally, combined remote sensing data from two time periods (9:30, 10:30, 11:30, and 12:30) on the 14th, 16th, and 18th were used, as well as remote sensing data from 9:30, 10:30, 11:30, and 12:30 on the 18th to create training samples. The resulting experimental data are referred to as DS1-RT, DS2-RT, DS3-RT, DS4-RT, and DS5-RT data, respectively. Remote sensing data from 13:30 on the 18th were selected as the test sample. As shown in Figure 2, the red tide index of the study area’s test set is visualized. Furthermore, considering computer memory limitations, the input sample size for the model was set to 32 × 32, and the images and corresponding labels were randomly divided into 32 × 32 pixel sample images with a stride of 4.

### 2.3. Relevant Methods

#### 2.3.1. U-Net Model

U-Net is a convolutional neural network (CNN) architecture originally designed to address medical image segmentation problems [17]. The network functions as a pixel-level prediction model that can be trained end-to-end, meaning it directly outputs segmentation maps from raw images. This training approach allows the model to excel in tasks such as medical image segmentation and is also well-suited for red tide detection. Through end-to-end training, the U-Net network can learn the direct mapping relationship from input images to red tide areas. Its structural advantage over other networks lies in its ability to train models with limited data, making it effective for small-sample image segmentation tasks. The skip connections in U-Net’s encoder–decoder architecture enable the fusion of features from different layers. In red tide detection, these skip connections help the network better recover and locate red tide areas, achieving higher segmentation accuracy. Therefore, research on red tide detection has been conducted based on the U-Net model.

The overall network structure of U-Net can be divided into two parts: the downsampling network and the upsampling network. The downsampling network reduces the resolution and size of the image through successive convolution and pooling layers, extracting features layer by layer. The upsampling network, on the other hand, restores the low-resolution image to its original size through deconvolution and skip connections. In red tide detection, this structure helps the network integrate the features of red tides. Building on the traditional encoder–decoder architecture, the concept of long connections is introduced, where feature maps from the downsampling process are directly transmitted to the upsampling process, thereby preserving more spatial information. This connection method allows the upsampling process to recover red tide remote sensing image information while retaining more details and edge information, enabling the reuse of shallow local features in the deeper layers of the network and significantly improving the accuracy of semantic segmentation. The feature extraction and recovery capabilities of the U-Net network enable it to precisely locate red tide areas and output high-quality segmentation results. In the final output layer, each pixel is assigned a category label indicating whether it belongs to a red tide area. Feature maps, which are intermediate results obtained by applying a series of convolution kernels to the input image, represent the extracted features from the original input image during the deep learning process. By acquiring features at various levels through the red tide detection model, the final red tide detection is achieved.

#### 2.3.2. Comparison Method

For comparison, this study selected the fully convolutional network (FCN) and a machine learning method, support vector machine (SVM). The details of each algorithm are as follows:

Fully convolutional networks (FCN), proposed by Jonathan Long et al. in 2015, is a framework for image semantic segmentation and is considered a pioneering work in applying deep learning to the field of semantic segmentation [18]. FCN replaces the fully connected layers in traditional CNNs with convolutional layers [19]. Additionally, to address the reduction in image size caused by convolution and pooling, FCN uses upsampling to restore the image size. The FCN network structure is mainly divided into two parts: the fully convolutional part and the deconvolutional part. The fully convolutional part is used for feature extraction, while the deconvolutional part restores the original size of the semantic segmentation image through upsampling. FCN includes three networks: FCN-32s, FCN-16s, and FCN-8s. FCN-32s restores the input size from 32x downsampled feature maps, while FCN-16s and FCN-8s restore the input size from 16x and 8x downsampling, respectively. FCN-8s integrates information from max-pooling layers and uses more deconvolutional layers for upsampling, resulting in finer segmentation. Therefore, FCN-8s outperforms FCN-16s and FCN-32s in image segmentation. FCN-8s is a deep learning model for image segmentation, primarily divided into fully convolutional and deconvolutional parts, where the fully convolutional part extracts features, and the deconvolutional part restores the original size of the semantic segmentation image through upsampling. Hence, FCN-8s was selected for comparative experiments [20].

SVM is a kernel-based supervised classification algorithm proposed by Cortes and Vapnik [21]. SVM is primarily a method for learning, classifying, and predicting small sample data, and it exhibits strong generalization capabilities. It has been proven to be a powerful machine learning algorithm for pattern recognition and nonlinear regression, offering significant advantages in the field of classification. The basic idea of SVM is to find the optimal hyperplane that can correctly divide the training dataset with the maximum geometric margin, where one side of the hyperplane represents red tide and the other side represents non-red tide [22].

## 3. CSF-RTDNet Red Tide Detection Model

### 3.1. Flowchart of Red Tide Detection Based on the CSF-RTDNet Model

**Figure 3:**
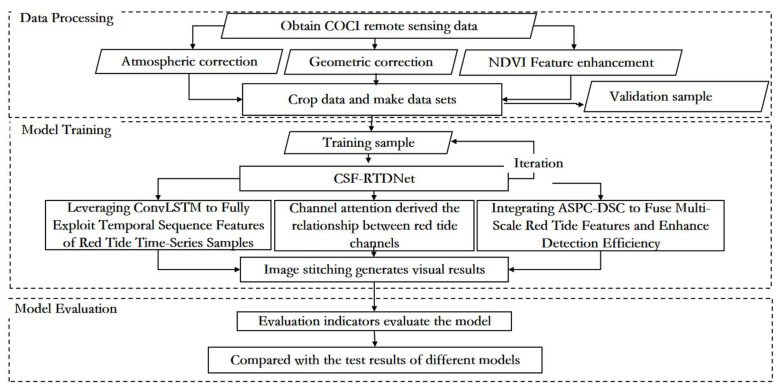
Overall flowchart of the CSF-RTDNet model.

The overall technical approach of this paper is shown in Figure 3, which is mainly divided into three parts: data processing, model training, and model evaluation. Firstly, the L1B data from the GOCI satellite is preprocessed through geometric correction, atmospheric correction, and other data processing methods, and the NDVI feature is fused to enhance the characteristics of red tides. Then, the data are cropped to create a dataset. Model training involves inputting the processed dataset into the red tide detection model based on the time series fusion network model for training. The red tide detection method based on the time series fusion network model introduces the ECA channel attention mechanism module, the atrous spatial pyramid convolution and depthwise separable convolution fusion module (ASPC-DSC), and the dropout layer on the basis of the traditional U-Net. This allows the model to effectively achieve cross-channel interaction, assign different weights according to the influence of different channels, and aggregate multi-scale contextual red tide feature information under multiple sampling rates, reducing computational resource usage while enhancing the ability to capture spatial information and improving the computational efficiency and inference speed of the model. The introduction of the convolutional long short-term memory network (ConvLSTM) effectively integrates the spatiotemporal feature changes of red tides at different time periods, fully exploiting the time series features of red tide samples. This enables better detection of red tides, and the relevant parameters are saved after the model is trained. Finally, the model evaluation uses metrics such as accuracy, precision, recall, and the kappa coefficient to assess the detection performance of the model and compares it with other red tide detection methods such as the support vector machine (SVM) model, the fully convolutional neural network (FCN), and the basic U-Net network model.

### 3.2. Feature Enhancement—NDVI

During a red tide event, microalgae, protozoa, or bacteria in the water undergo explosive proliferation or aggregation under certain environmental conditions, leading to an increase in chlorophyll content and changes in the water’s spectral characteristics. In GOCI remote sensing images, significant changes occur in the original six bands (412, 443, 490, 555, 660, and 680 nm), with the most pronounced fluctuations observed in the blue and green bands.

Due to the small spectral differences between pixels at the boundaries of red tides, the separability between red tides and seawater is weak, making it difficult to accurately distinguish the complex boundaries of small-scale, dispersed, and strip-like red tides. Research shows that high-resolution remote sensing images contain complex and rich red tide feature information, with strong responses in the red and near-infrared bands. The NDVI (Normalized Difference Vegetation Index) of red tides significantly differs from that of seawater and other objects. Therefore, this paper introduces NDVI as an input feature. The average NDVI values of both red tides and seawater are less than 0, with seawater having the smallest NDVI value, followed by red tides. Introducing NDVI enhances the feature information of red tides, making it easier to distinguish small-scale, dispersed, and strip-like boundary red tides. By combining GOCI’s original six-band data with NDVI, the spectral differences between red tides and seawater can be increased, enhancing their separability. In GOCI band data, the red and near-infrared bands correspond to B5 and B8, respectively. The NDVI Formula (2) is as follows:


(2) $$NDVI=\frac{R_{rs}\left(865\right)-R_{rs}\left(660\right)}{R_{rs}\left(865\right)+R_{rs}\left(660\right)}$$

### 3.3. Attention Mechanism of ECA Channel

The basic idea of attention mechanisms is to compute an importance distribution over the model’s inputs (such as word sequences or different regions of an image) and then weight the inputs based on this distribution, allowing the model to focus on more useful information. This is typically achieved by calculating a weighted sum of input features, where the weights are dynamically learned by the model and reflect the importance of each feature to the current task. Research shows that red tides are certain to occur in high-chlorophyll waters and may occur in low-chlorophyll waters. When the chlorophyll content in the water increases, red tides also erupt, and the data in the blue and green bands of remote sensing images show significant changes. Therefore, in red tide detection, assigning different weights based on the importance of different bands to red tide detection can effectively improve the classification accuracy of the detection model.

**Figure 4:**
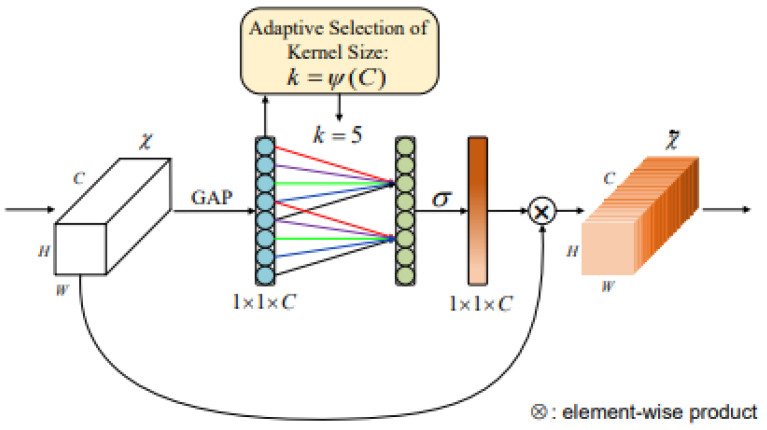
ECA module.

The ECA module can effectively achieve cross-channel interaction, assigning different weights based on the influence of each channel, thereby improving the classification accuracy of the detection model. Assuming an input feature map of size H × W × C, global pooling (with a pooling size of H × W) is first applied to obtain a 1 × 1 × C feature map, retaining only the channel dimension. This is followed by a 1D convolutional layer, enabling information interaction between each channel and its adjacent channels while sharing weights. Subsequently, a sigmoid layer is connected to produce a 1 × 1 × C feature. Finally, the original H × W × C feature map is multiplied element-wise with the 1 × 1 × C feature map to obtain the feature map enhanced with attention weights. By performing element-wise multiplication on the feature map, attention weights are increased, adjusting the correlation between different channels in the feature map and enhancing the model’s accuracy [23]. The specific structure is illustrated in Figure 4:

The ECA module structure is color-coded as follows: light blue represents the compressed features after global average pooling (GAP), green denotes the adaptive selection of 1D convolution kernel size for local cross-channel interaction, and orange indicates the final channel attention weights generated via Sigmoid activation. Colored arrows visualize dynamic interaction paths between channels, while brown highlights the output features recalibrated by attention weights. 

### 3.4. Atrous Spatial Pyramid Convolution and Depthwise Separable Convolution (ASPC-DSC)

Atrous spatial pyramid pooling (ASPP) is a convolutional neural network structure in deep learning that combines the concepts of atrous convolution and spatial pyramid pooling [24]. It is primarily used to process image data with multi-scale information and has demonstrated significant effectiveness. ASPP performs parallel sampling on the given input using atrous convolutions with different sampling rates to achieve pooling operations. It is then combined with global average pooling to form a new feature pyramid model, thereby aggregating multi-scale contextual information. This approach leverages atrous convolution to expand the receptive field of the convolutional kernel without downsampling. Specifically, atrous convolutions with rates of 1, 3, and 5 and a kernel size of 33 are employed, and depthwise separable convolution is used to reduce the number of parameters. The specific implementation of the ASPP module is as follows: the first branch uses a 11 standard convolution to maintain the original receptive field; the second to fourth branches use depthwise separable convolutions with different dilation rates to extract features and obtain varying receptive fields; and the fifth branch applies global average pooling to the input to capture global features. Finally, the feature maps from the five branches are concatenated along the channel dimension, and a 1 × 1 standard convolution is used to fuse the multi-scale information.

**Figure 5:**
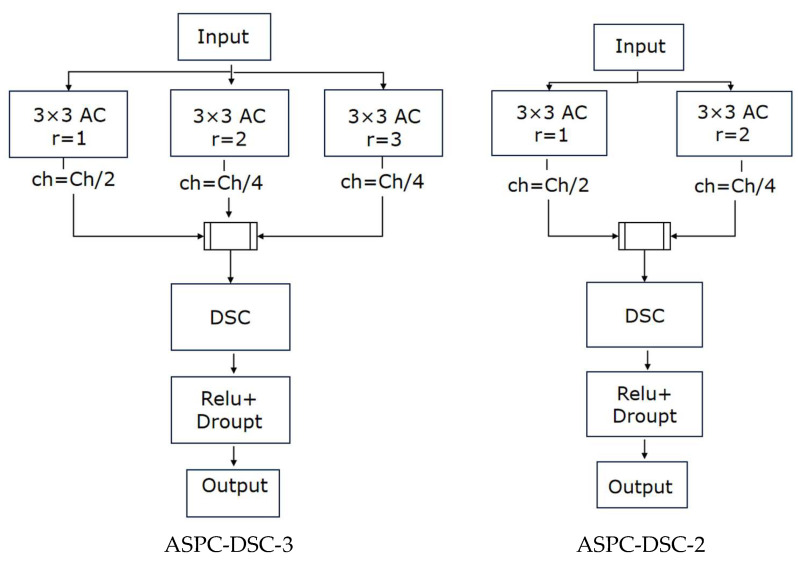
ASPC-DSC module.

In this paper, inspired by the concept of ASPP, we propose the atrous spatial pyramid convolution with depthwise separable convolution (ASPC-DSC) module. ASPC consists of multiple parallel convolutions with different dilation rates, forming a new feature pyramid model that expands the receptive field of the convolutional kernel, thereby aggregating multi-scale contextual information. This enhances the model’s ability to extract features of red tides with complex boundaries at different scales, improving its capability to detect small-scale, dispersed, and strip-like red tides. Building on ASPC, ASPC-DSC introduces depthwise separable convolution (DSC) [25], creating a novel and efficient feature extraction module. This module not only expands the receptive field and aggregates multi-scale contextual information, enhancing the model’s ability to detect red tide regions with complex boundaries and varying scales, but also reduces computational complexity through DSC, improving detection efficiency. As a result, it optimizes the model’s inference speed without compromising detection accuracy, thereby enhancing overall detection performance. The ASPC-DSC module adopts a three-branch parallel atrous convolution structure, where ASPC-DSC-3 uses dilation rates of 1, 2, and 3, with channel proportions of 50%, 25%, and 25%, respectively, and ASPC-DSC-2 uses dilation rates of 1 and 2, with channel proportions of 50% and 50%, respectively. As the number of convolutional layers increases, ASPC-DSC exhibits the property of a linearly growing receptive field with the dilation rate. For example, with a dilation rate of 2, the receptive field of a 3 × 3 convolutional kernel increases to 7 × 7, and with a dilation rate of 3, it increases to 11 × 11, ensuring the model can aggregate multi-scale contextual red tide feature information. Additionally, the integrated depthwise separable convolution (DSC) further optimizes the feature extraction method on the basis of ASPC, enabling the model to enhance its ability to capture spatial information while reducing computational resource usage, thereby improving red tide detection accuracy. The specific structure is illustrated in Figure 5:

### 3.5. Long Short-Term Memory Network (LSTM)

Long short-term memory (LSTM) is a special type of recurrent neural network (RNN) architecture. LSTM is designed to address the issues of vanishing and exploding gradients that traditional RNNs encounter when processing long sequence data, enabling the network to learn and maintain long-term dependencies. The main feature of LSTM is the introduction of gate mechanisms and memory cells, which allow for the storage and control of long-term information. Specifically, the forget gate determines which historical information should be discarded, the input gate decides which new information should be stored in the memory cell, the reset gate determines which memories should be reset, and the output gate controls the output of the memory cell. This mechanism enables LSTM to retain long-term information and utilize it for prediction and decision-making when needed [26].

LSTM has achieved successful applications in many fields, including speech recognition, image captioning, and natural language processing. For example, in speech recognition, LSTM can handle long-term dependencies in speech signals, thereby improving the accuracy of speech recognition. In image captioning, LSTM can generate descriptive text that matches the content of the image. In natural language processing, LSTM can manage long-term dependencies within sentences or paragraphs, enhancing the performance of tasks such as text classification and sentiment analysis. LSTM is a highly effective deep learning model capable of addressing long-term dependencies in sequential data and has been successfully applied in numerous domains.

### 3.6. CSF-RTDNet Model Framework

**Figure 6:**
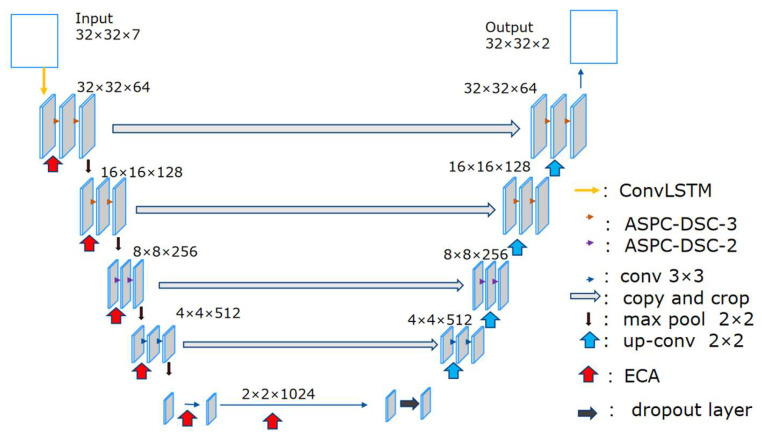
CSF-RTDNet model structure.

Through the study of previous red tide detection models, it has been found that traditional red tide detection methods, which rely on expert experience and qualitative analysis based on extensive reports, often have inaccurate thresholds and poor applicability. Additionally, the features used are relatively singular and insufficiently rich, limiting the improvement of red tide detection accuracy. Due to the strong temporal continuity and correlation in the development of red tides, current U-Net-based red tide detection methods can effectively detect red tides on the day they occur but perform poorly in detecting red tides in the following days. This is due to insufficient mining of temporal sequence features in time-series samples, which hinders better red tide detection. Therefore, based on the traditional U-Net, the ECA module is introduced to assign different weights according to the influence of different bands, allowing the model to focus on the channels that require the most attention, thereby improving the classification accuracy of the detection model. Simultaneously, the ordinary convolutional blocks in the first two layers of the U-Net structure are replaced with the ASPC-DSC-3 module, and the ordinary convolutional block in the third layer of the U-Net structure is replaced with the ASPC-DSC-2 module. This enables the fusion of multi-scale contextual red tide feature information under multiple sampling rates, enhancing the model’s ability to extract features at different scales. Depthwise separable convolution further optimizes the feature extraction method, allowing the model to enhance its ability to capture spatial information while reducing computational resource usage, thereby improving the model’s computational efficiency and inference speed. While maintaining multi-scale perception capabilities, the receptive field is expanded, strengthening the extraction of red tide features in complex boundary regions and improving red tide detection accuracy. The introduction of convolutional long short-term memory (ConvLSTM), which combines the temporal sequence processing capability of LSTM and the spatial feature processing capability of CNN, effectively integrates the spatiotemporal feature changes of red tides across different time periods. This fully exploits the temporal sequence features of red tide time-series samples, enabling more precise identification of red tides and thereby improving the classification accuracy of the detection model. Additionally, since the model training in this study is based on a small training set, a dropout layer is added before upsampling to avoid overfitting, which to some extent reduces the information lost during downsampling. The overall structure of the improved red tide detection model proposed in this paper is shown in Figure 6.

## 4. Experimental Results and Analysis

### 4.1. Experimental Settings

The experiments in this paper were conducted on a device with the following specifications: Intel Core i7-11700, 2.50 GHz processor, and 16 GB RAM. All hardware components were commercially available products from their respective manufacturers. The entire experiment was implemented using the Python 3.8.10 and TensorFlow 2.6.0 frameworks. For the CSF-RTDNet model, the input size is 32 × 32 with seven channels, and the output is a classification image of the same size. In the experiments, the Adam optimization algorithm was used for the model [27], with a batch size of 50, a dropout rate of 0.3, and 120 training epochs.

### 4.2. Model Evaluation Index

To comprehensively evaluate the classification accuracy of the model, this paper introduces accuracy, precision, recall, and the kappa coefficient as evaluation metrics.

Accuracy represents the proportion of samples where positive samples are correctly classified as positive and negative samples are correctly classified as negative, out of the total number of samples. Formula (3) is as follows:

(3) $$Accuracy=\frac{TP+TN}{TP+TN+FP+FN}\times 100\%$$

Precision is the ratio of accurately detected red tide pixels to all identified red tide pixels. Formula (4) is as follows:

(4) $$Precision=\frac{TP}{TP+FP}\times 100\%$$

Recall represents the percentage of red tide pixels that are correctly classified. Formula (5) is as follows:

(5) $$Recall=\frac{TP}{TP+FN}\times 100\%$$

The kappa coefficient is an accuracy evaluation metric used to measure the consistency of red tide detection results with greater objectivity. Formula (6) is as follows:

(6) $$Kappa=\frac{TP+TN}{\left(TN+FP)\times (TN+FN)+(FN+TP)\times (FP+TP\right)}\times 100\%$$

Among them, true positive (TP): the number of pixels that are actually red tide and are also detected as red tide; false positive (FP): the number of pixels that are actually non-red tide but are misdetected as red tide; true negative (TN): the number of pixels that are actually non-red tide and are also detected as non-red tide; and false negative (FN): the number of pixels that are actually red tide but are detected as non-red tide.

### 4.3. Experimental Results

#### 4.3.1. Comparative Analysis of Experimental Results for Different Samples

Table 2 presents the red tide detection results comparing the remote sensing data samples DS1-RT from 13:30 on 14 to 17 August 2020, the remote sensing data sample DS5-RT from the 18th, and the combined data sample DS4-RT of DS1-RT and DS5-RT, using the ECA channel attention mechanism and the atrous spatial pyramid convolution (ASPC) model algorithm based on the traditional U-Net. In terms of accuracy, precision, recall, and the kappa coefficient, the combined remote sensing data from the two time periods achieved better red tide detection results, with improvements in accuracy, precision, recall, and the kappa coefficient. This indicates that remote sensing data samples from the same time point on different days and combined remote sensing data samples from different time points on the same day contain more red tide feature information, leading to more effective red tide detection.

#### 4.3.2. Comparative Analysis of Model Validation Experimental Results

Table 3 presents the classification results comparing the traditional U-Net with the introduction of the ECA channel attention mechanism and atrous spatial pyramid convolution (ASPC), the ConvLSTM module, and the ASPC-DSC module, using remote sensing data samples from the same time point on different days (14th to 17th) and combined remote sensing data samples from different time points on the 18th (DS4-RT). In terms of accuracy, precision, recall, and the kappa coefficient, the red tide detection method based on the time series fusion network model achieved the best detection and classification results, with an accuracy of 95.89%, precision of 93.03%, recall of 96.34%, and a kappa coefficient of 0.95. This indicates that the red tide detection method based on the time series fusion network model first learns the spatiotemporal feature changes through ConvLSTM, and then, by introducing the ECA channel attention mechanism and the ASPC-DSC module (atrous spatial pyramid convolution with depthwise separable convolution), effectively fuses spatial features at different scales. This allows for better, faster, and more efficient detection of red tide pixels, resulting in higher red tide detection accuracy. The specific analysis is as follows:

Compared to the model algorithm that introduces the ECA channel attention mechanism and atrous spatial pyramid convolution (ASPC) based on the traditional U-Net, the model that incorporates the ECA channel attention mechanism, ConvLSTM module, and ASPC-DSC module shows improvements in accuracy, precision, recall, and the kappa coefficient by 4.48%, 6.09%, 5.65%, and 0.06, respectively. The model algorithm based on the traditional U-Net with the ECA channel attention mechanism and ASPC can effectively extract red tide feature information and perform effective detection of red tides on the same day. However, its performance in detecting red tides in the following days is not ideal. This is because, from the onset to the end of a red tide, the collection of temporally continuous red tide samples is insufficiently mined for temporal sequence features by the model algorithm based on the traditional U-Net with the ECA channel attention mechanism and ASPC, thus limiting its ability to better detect red tides. The introduced atrous spatial pyramid convolution with depthwise separable convolution (ASPC-DSC) module fuses multi-scale contextual red tide feature information under multiple sampling rates, enhancing the model’s ability to extract features at different scales. Additionally, depthwise separable convolution further optimizes the feature extraction method, enabling the model to enhance its ability to capture spatial information while reducing computational resource usage, thereby improving the model’s computational efficiency and inference speed. While maintaining multi-scale perception capabilities, the receptive field is expanded, strengthening the extraction of red tide features in complex boundary regions and improving the accuracy of red tide detection.

The introduced ConvLSTM is an architecture that combines convolutional neural networks (CNN) and long short-term memory (LSTM) networks, possessing both the temporal sequence processing capability of LSTM and the spatial feature processing capability of CNN. It is specifically designed to handle sequential data. Unlike traditional LSTM, ConvLSTM incorporates convolutional structures into the input gate, output gate, and forget gate of the LSTM to simultaneously capture spatiotemporal features. This helps in capturing spatial information in sequential data and fully exploiting the temporal sequence features of red tide time-series samples, resulting in higher accuracy in red tide detection.

#### 4.3.3. Comparative Analysis of Detection Results Incorporating Different Temporal Data

Table 4 presents the red tide detection results comparing different temporal data using the CSF-RTDNet model. In terms of accuracy, precision, recall, and the kappa coefficient, the combined remote sensing data DS4-RT from the 18th and the same time point on different days achieved the best red tide detection results, with an accuracy of 95.89%, precision of 93.03%, recall of 96.34%, and a kappa coefficient of 0.95. Meanwhile, the remote sensing data DS1-RT from 13:30 on the 14th to 17th also achieved good detection results, with an accuracy of 89.98%, precision of 91.70%, recall of 80.27%, and a kappa coefficient of 0.85. Further analysis shows that the combined remote sensing data DS2-RT from the 14th and 13:30 on the 14th to the 17th improved accuracy, precision, recall, and the kappa coefficient by 1.45%, 18.54%, 4.90%, and 0.11, respectively, compared to DS1-RT. These results indicate that the combined remote sensing data DS4-RT from the 18th and the same time point on different days contains more red tide feature information, while the remote sensing data DS1-RT from 13:30 on the 14th to the 17th, through the CSF-RTDNet model algorithm, fully exploits the temporal sequence features of red tide samples and effectively integrates spatial features at different scales. The fusion of multi-temporal data, combining same-time-point data from previous days and multi-time-point data from the same day (DS4-RT), captures richer spatiotemporal features. By leveraging the spatiotemporal feature extraction capability of ConvLSTM, the ECA channel attention mechanism, the fusion of deep and shallow features in ASPC-DSC, and the efficient computation of multi-scale spatial features, the method effectively addresses the issue of insufficient temporal feature mining, achieving higher red tide detection accuracy and greater practical significance.

#### 4.3.4. Comparative Analysis of Experimental Results from Different Model Methods

Using remote sensing data samples from the same time point on different days (14th to 17th) and the combined remote sensing data sample DS4-RT from different time points on the 18th, the CSF-RTDNet model algorithm was compared with the basic U-Net method, fully convolutional neural network (FCN), and support vector machine (SVM). The parameters of the FCN used in this experiment were the same as those of the CSF-RTDNet model algorithm. As shown in Table 5, the CSF-RTDNet model method, which incorporates the ECA channel attention mechanism, ASPC-DSC module, and ConvLSTM module, can fully exploit the temporal sequence features of red tide samples and effectively integrate spatial features at different scales, achieving the best red tide detection performance with an accuracy of 95.89%, precision of 93.03%, recall of 96.34%, and a kappa coefficient of 0.95. Compared to the machine learning method SVM, the CSF-RTDNet model method shows significant improvements in accuracy, precision, recall, and the kappa coefficient. Additionally, under the same training set and parameters, the CSF-RTDNet model method outperforms FCN-8s, with noticeable enhancements in accuracy, precision, recall, and the kappa coefficient. The CSF-RTDNet model method also surpasses the basic U-Net model, with improvements in accuracy, precision, recall, and the kappa coefficient by 9.37%, 9.72%, 17.07%, and 0.14, respectively. The experimental results demonstrate that the CSF-RTDNet model method is more suitable for red tide detection.

#### 4.3.5. Comparative Analysis of Visualization Results

**Figure 7:**
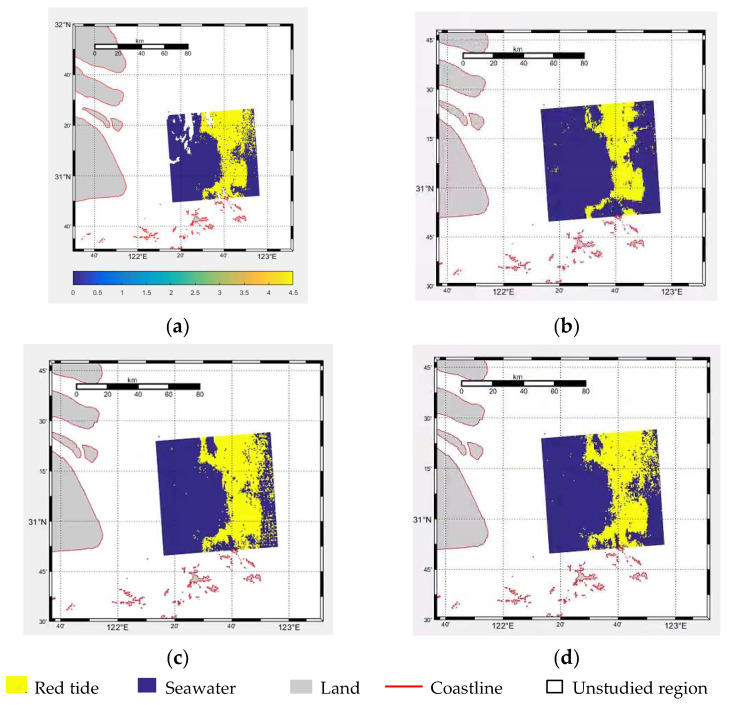
Red tide detection results based on different time series data. (**a**) Actual red tide occurrence area; (**b**) DS1-RT data detection result; (**c**) DS2-RT data detection result; (**d**) DS4-RT data detection result.

The CSF-RTDNet model algorithm can more effectively detect red tides. Figure 7 displays the visualization of the actual red tide occurrence area selected at 13:30 on the 18th (a), the detection results of the remote sensing data sample DS1-RT from the same time point on different days (14th to 17th) (b), the detection results of the combined data sample DS2-RT from the 14th (c), and the detection results of the combined data sample DS4-RT from the 18th (d). From the figures, it can be observed that the combined data sample DS4-RT from the 18th achieved the best red tide detection results using the improved U-Net model in this chapter, closely resembling the actual red tide occurrence area shown in Figure 7a. This demonstrates that the CSF-RTDNet model algorithm in this study, by integrating the spatiotemporal feature extraction capability of ConvLSTM, the ECA channel attention mechanism, the fusion of deep and shallow features in ASPC-DSC, and the efficient computation of multi-scale spatial features, effectively addresses the issue of insufficient temporal feature mining. It also indicates that the combined remote sensing data samples from the same time point on different days before the red tide event on the 18th and from different time points on the same day contain rich red tide feature information, enabling effective red tide detection and holding significant practical value.

## 5. Conclusions

The occurrence of red tides is related to various factors, including the physical and chemical conditions of the marine environment, the dynamic changes of biological populations, and meteorological conditions. For example, high nutrient concentrations, suitable water temperature and light conditions, and the proliferation of certain plankton populations can all contribute to the occurrence of red tides. Detecting red tides is challenging because their formation is a complex process involving the interaction of multiple factors. Additionally, the development of red tides exhibits strong temporal continuity and correlation. Current deep learning-based red tide detection methods often inadequately mine temporal sequence features, limiting their ability to effectively detect red tides. The CSF-RTDNet model algorithm proposed in this study addresses these challenges by integrating multi-temporal data, combining same-time-point data from previous days and multi-time-point data from the same day, to capture richer spatiotemporal features, thereby significantly improving red tide detection accuracy. Specifically, CSF-RTDNet first uses ConvLSTM to learn the spatiotemporal feature changes of the input remote sensing data. ConvLSTM combines the temporal sequence processing capability of LSTM with the spatial feature processing capability of CNN, effectively integrating the spatiotemporal feature changes of red tides across different time periods and fully exploiting the temporal sequence features of red tide samples. Furthermore, the ECA channel attention mechanism is introduced to assign different weights based on the influence of different bands on red tide detection, enabling deeper extraction of red tide features. The atrous spatial pyramid convolution with depthwise separable convolution (ASPC-DSC) module is designed to fuse multi-scale contextual red tide feature information under multiple sampling rates, enhancing the model’s ability to extract features at different scales. Depthwise separable convolution further optimizes the feature extraction process, allowing the model to enhance its ability to capture spatial information while reducing computational resource usage, thereby improving computational efficiency and inference speed. By integrating deep and shallow features as well as multi-scale spatial features, the model effectively addresses the issue of insufficient mining of temporal sequence features, significantly improving red tide detection accuracy.

Red tide detection is of great significance for prevention and management. By detecting the occurrence of red tides, timely measures can be taken, such as reducing pollutant emissions and adjusting fishing activities, to mitigate the impact of red tides on marine ecosystems and human health. This study explores the application of GOCI remote sensing data in red tide detection, and the experimental results demonstrate that the proposed method achieves promising detection performance, providing a new reference for red tide detection methods.

## Figures and Tables

**Table 1:** Average solar irradiance outside the atmosphere at each band of GOCI.

Band Center (nm)	Solar Irradiance $(W\cdot m^{-2}\cdot \mu m^{-1}\cdot sr^{-1}$)
412	1738. 8
443	1922. 1
490	1988. 4
555	1869. 9
660	1535. 3
680	1508. 3
745	1295. 9
865	967. 6

**Table 2:** Comparison of red tide detection accuracy for different samples.

Data	Accuracy/(%)	Precision/(%)	Recall/(%)	Kappa
DS1-RT	85.93	86.96	73.32	0.80
DS5-RT	88.26	84.10	84.58	0.84
DS4-RT	91.41	86.94	90.69	0.89

**Table 3:** Comparison of red tide detection accuracy with different mechanisms introduced in U-Net.

Method	Accuracy/(%)	Precision/(%)	Recall/(%)	Kappa
Basic U-Net	86.52	83.76	79.27	0.81
U-Net + ECA + ASPC-DSC	91.41	86.94	90.69	0.89
U-Net + ECA + ASPC-DSC + ConvLSTM	95.89	93.03	96.34	0.95

**Table 4:** Comparison of red tide detection accuracy across different samples.

Method	Accuracy/(%)	Precision/(%)	Recall/(%)	Kappa
DS1-RT	89.98	91.70	80.27	0.85
DS2-RT	92.55	91.91	87.63	0.90
DS3-RT	91.85	90.66	84.00	0.87
DS4-RT	95.89	93.03	96.34	0.95

**Table 5:** Comparison of red tide detection accuracy across different methods.

Method	Accuracy/(%)	Precision/(%)	Recall/(%)	Kappa
SVM	77.92	91.35	42.48	0.57
FCN-8s	83.54	93.82	45.02	0.61
Basic U-Net	86.52	83.76	79.27	0.81
CSF-RTDNet	95.89	93.03	96.34	0.95

## Data Availability

Inquiries regarding the experimental data should be made by contacting the first author.
